# Primary Ankle Fracture Dislocation Is Not a Negative Prognostic Factor for the Surgical Treatment of Syndesmotic Injury—A Retrospective Analysis of 246 Patients

**DOI:** 10.3390/jcm14041215

**Published:** 2025-02-12

**Authors:** Błażej Grzegorz Wójtowicz, Katarzyna Chawrylak, Jędrzej Lesman, Hubert Makowski, Kacper Kuczyński, Michał Maciejowski, Alicja Majos, Marcin Domżalski

**Affiliations:** 1 Department of Orthopedics and Trauma, Medical University of Lodz, Veteran’s Memorial Hospital, Zeromskiego 113 St., 90-549 Lodz, Poland; jedreklesman@yahoo.pl (J.L.); marcin.domzalski@umed.lodz.pl (M.D.); 2 Department of Surgical Oncology, Medical University of Lublin, Radziwiłłowska 13 St., 20-080 Lublin, Poland; katchawrylak@gmail.com; 3 Student Scientific Group, Department of Orthopedics and Trauma Medical University of Lodz, Al. Tadeusza Kościuszki 4, 90-419 Lodz, Poland; hubert.makowski@stud.umed.lodz.pl (H.M.); kacper.kuczynski@stud.umed.lodz.pl (K.K.); michal.maciejowski@stud.umed.lodz.pl (M.M.); 4 General and Trasplant Surgery Department, Medical University of Lodz, Al. Tadeusza Kościuszki 4, 90-419 Lodz, Poland; alicja.majos@umed.lodz.pl

**Keywords:** ankle fracture, syndesmotic injury, tibiofibular syndesmosis, dislocation, open reduction and internal fixation

## Abstract

**Background/Objectives:** Acute ankle sprains are common injuries that significantly affect both sports and daily activities. Syndesmotic injuries, a specific type of ligamentous damage, can occur as a part of a sprain or alongside fractures, affecting approximately 20% of ankle fractures. The aim of this study was to evaluate negative prognostic factors influencing surgical outcomes in tibiofibular syndesmotic injuries associated with ankle fractures. **Methods:** Data from 246 patients were analyzed to examine the impact of initial ankle dislocation, fracture type, and fixation method on postoperative complications and reoperation rates. Ankle joint fractures were treated with open reduction and internal fixation using an anatomically contoured plate designed for optimal tibia and fibula fixation. Four methods of syndesmosis fixation were recorded: one three-cortical screw, one four-cortical screw, two screws (either both four-cortex screws or one three-cortex and one four-cortex screw), or one endobutton. Data analysis was performed using SPSS version 25 (IBM Corp., Armonk, NY, USA). **Results:** Key findings reveal no significant association between initial dislocation and the necessity for reoperation (*p* = 0.613). However, smoking combined with dislocation significantly increases reoperation rates (35% vs. 15.5%, *p* = 0.026). Fixation type influenced outcomes, with single four-cortex screws linked to pain but fewer infections. Infection was the most common complication (33.3%), predominantly after fixation with a single three-cortex screw. Men had higher rates of fixation destabilization and infections, while women experienced pain persisting beyond six months postoperatively **Conclusions:** Patient-specific factors influence syndesmotic injury outcomes. Smoking, gender, and fixation type impact complications, emphasizing the need for tailored surgical approaches to enhance recovery and minimize reoperation risks. Future research should aim to corroborate these findings in larger, multicentric cohorts to refine surgical strategies for syndesmotic injury management.

## 1. Introduction

Acute ankle sprains are common injuries that significantly impact both sports and daily life. In the United States, approximately 2 million acute ankle sprains occur annually, with emergency departments reporting an incidence rate of 2 to 7 per 1000 person-years. However, this number may be an underestimate, as many cases go unreported, suggesting the actual rate could be much higher [1]. Certain demographics are at a higher risk: females experience a higher incidence rate (13.6 per 1000 exposures) compared to males (6.9 per 1000 exposures), and children have a higher rate of sprains than adults [1,2].

Syndesmotic injuries, a specific type of ligamentous damage, can occur as a part of a sprain or alongside fractures. Approximately 20% of ankle fractures involve a syndesmotic injury, with an even higher incidence in rotational injury mechanisms. Athletes experience syndesmotic injuries in up to 25% of ankle sprains, with around 6500 cases reported annually in emergency rooms. Misdiagnosis can lead to chronic pain, dysfunction, and often require surgical intervention. Poor outcomes, including symptomatic osteoarthritis, can occur if the injury is not properly treated. Accurate diagnosis and appropriate treatment are crucial for restoring function and preventing long-term issues [3].

The tibiofibular syndesmosis consists of four primary ligaments: the interosseous membrane, the transverse ligament, the anterior inferior tibiofibular ligament (AITFL), and posterior inferior tibiofibular ligament (PITFL) [3]. The surgical treatment of syndesmotic injuries aims to restore the ankle’s normal anatomy and maintain stability. Various treatment options are available, each with distinct advantages [4]. Syndesmotic screws, a traditional method, involve inserting one or more screws across the syndesmosis to maintain reduction, which may be left in place or removed after healing, typically around 3–4 months post-surgery [5]. Suture button devices offer flexible fixation that allows physiological micromotion, promoting healing and resulting in fewer complications related to screw breakage and the need for removal [6]. Some protocols advocate for combination fixation, using both screws and suture buttons to optimize stability, particularly in severe injuries. Bioabsorbable implants provide temporary fixation, eliminating the need for a second surgery for removal, although their long-term outcomes are still being studied [6,7]. In cases where syndesmotic injury is associated with fractures, open reduction and internal fixation (ORIF) are performed to address the fractures while stabilizing the syndesmosis [8].

**Figure 1:**
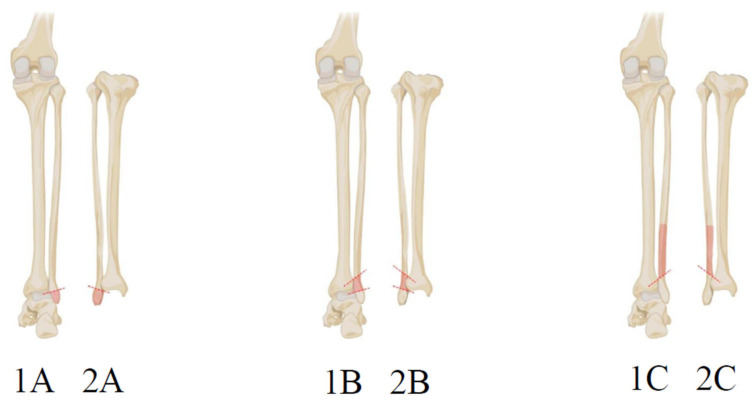
Danis–Weber classification of ankle fractures: type A posterior view (**1A**); type A anterior view (**2A**); type B posterior view (**1B**); type B anterior view (**2B**); type C posterior view (**1C**); and type C anterior view (**2C**).

The Weber classification system categorizes ankle fractures based on their location relative to the syndesmosis, which is the ligamentous connection between the tibia and fibula (Figure 1) [9].

Type A fractures occur below the level of the syndesmosis (infra-syndesmotic) and are typically transverse. In these fractures, the tibiofibular syndesmosis and the deltoid ligament remain intact. These fractures are usually stable, and treatment generally involves immobilization, walking with crutches and non-weightbearing for six weeks [10]. Type B fractures are located at the level of the syndesmosis (trans-syndesmotic) and may extend proximally. They usually present as spiral fractures. The tibiofibular syndesmosis is injured in about 50% of cases. The medial malleolus may be fractured, and the deltoid ligament may be torn, as indicated by a widening of the space between the medial malleolus and the talar dome. Stability varies based on the condition of the medial structures (malleolus or deltoid ligament) and the syndesmosis. These fractures mostly require ORIF [8]. Type C fractures occur above the level of the syndesmosis (supra-syndesmotic) and are characterized by the disruption of the tibiofibular syndesmosis with the widening of the distal tibiofibular articulation. A fracture of the medial malleolus or injury to the deltoid ligament is often present. These fractures can extend as proximally as the fibular neck and may not be visible on ankle X-rays, necessitating knee or full-length tibia–fibula radiographs to identify a Maisonneuve fracture. Type C fractures are generally unstable and usually require ORIF. Fractures above the syndesmosis typically result from external rotation or abduction forces that also disrupt the joint, often accompanied by an injury to the medial side [8,11].

Several factors impact the prognosis of surgical treatment for syndesmotic injuries. The accuracy of anatomical reduction is crucial for achieving optimal outcomes, as malreduction can result in chronic pain and dysfunction. The choice of fixation technique plays a significant role in the overall success of the treatment [6,7]. Additionally, patient-specific factors such as advanced age, low activity levels, and comorbid conditions like diabetes, obesity, and smoking increase infection risk, prolong healing, and contribute to chronic pain [12].

The primary objective of this study was to investigate whether the initial dislocation of the ankle joint with malleolar fractures had influence on subsequent complications following tibiofibular syndesmosis fixation. We also looked for other potential negative prognostic factors such as the type of fracture, the fixation method, gender, age, and smoking habits.

## 2. Materials and Methods

### 2.1. Study Design

This retrospective observational study analyzed patients with ankle fractures admitted to the Department of Orthopedics and Trauma from 1 January 2016 to 1 December 2023. The electronic patient database was queried to identify all patients who underwent surgical treatment for malleolar ankle fractures or isolated syndesmotic injuries using a specific operation code. The decision for operative treatment (including qualification for surgery and choice of fixation method) was made by the trauma surgeon on duty, a board-certified orthopedic trauma specialist at a high-referral trauma center. The authors confirm that all methods were performed in accordance with relevant guidelines, following the hospital’s orthopedic and trauma protocols, which are based on national trauma surgery guidelines. Due to the study’s retrospective nature, approval from the Internal Reviewing Board was not required according to national regulations Dz. U. 2021r. poz.790, 1559.

### 2.2. Setting

Baseline characteristics collected included age at the time of surgery, gender, smoking, observation period, reoperation, and the purpose of reoperation. Injuries were classified according to the Weber classification or as isolated syndesmosis injuries, with further categorization based on the presence of dislocation. The method of tibiofibular syndesmosis fixation (1 three-cortical screw, 1 four-cortical screw, 2 screws, and 1 suturebutton) was assessed using preoperative and postoperative radiographic images, such as Computed Tomography (CT) scans and X-rays of the injured ankle. Reoperations were defined as procedures directly related to tibiofibular syndesmosis fixation, including syndesmosis screw removal or screw exchange.

### 2.3. Inclusion and Exclusion Criteria

The study included adults aged 18 years and older who underwent surgical fixation for tibiofibular syndesmosis injuries and later required reoperation. Patients were excluded if they were under 18, had no syndesmotic injury, or had their initial surgery performed at a different center. Pilon and tibial shaft fractures were excluded due to their distinct injury mechanisms, which could influence complication rates. Patients routinely undergoing syndesmosis screw removal or those with an observation period of less than 8 weeks—the minimum time required for bone healing—were also excluded. Additionally, individuals with incomplete medical records, those who did not follow-up for at least 12 months, or those with concomitant lower limb injuries that could affect outcomes were not included. Since this was a retrospective study, follow-up duration varied, depending on the availability of patient records in the outpatient clinic. No patients with severe peripheral vascular disease or neuropathies were identified in the department.

### 2.4. Quantitative Variables

Initially, 846 patients with tibia or fibula fractures and isolated syndesmotic injuries were identified. Exclusions were made as follows: 2 patients due to age under 18, 54 patients due to pilon fractures, 150 patients due to tibial shaft fractures, 105 patients due to the absence of syndesmosis injury, 130 patients due to conservative (non-surgical, used in cases deemed stable without surgical fixation, patients unfit for surgery, or those refusing surgery) treatment, 77 patients due to a short observation period, and 84 patients due to the routine removal of the syndesmosis screw. Eventually, 246 patients were included in the study.

### 2.5. Surgical Technique

Ankle joint fractures were treated with ORIF using an anatomically contoured plate designed for optimal tibia and fibula fixation. Four methods of syndesmosis fixation were recorded, i.e., 1 three-cortical screw, 1 four-cortical screw, 2 screws, or 1 suturebutton. The choice of fixation method was determined by the trauma surgeon based on intraoperative findings and surgeon preference. The stability of the syndesmosis was evaluated intraoperatively using the Cotton test (the widening of the syndesmosis with lateral pull on the fibula) and fluoroscopic views. Initial reductions in ankle dislocations were performed in the emergency unit. Postoperatively, patients were immobilized for 4 to 6 weeks, depending on whether the injury was a fracture or an isolated syndesmosis injury.

### 2.6. Limitation of the Study

This study has several limitations inherent to its retrospective design. Over 70 patients were excluded due to a short observation period, and long-term functional outcomes, including quality of life and return to physical activity, were not assessed, limiting the broader applicability of the findings. Deep infections were classified based on cases requiring implant removal, and only diagnosed diabetes was recorded without a routine HbA1c assessment. Data collection focused primarily on smoking and dislocation due to their known impact on the reoperation risk, while other comorbidities were inconsistently reported, making data consolidation difficult. Additionally, fixation methods were chosen at the surgeon’s discretion rather than following a standardized protocol, introducing variability in treatment outcomes. The single-center design, which includes a high-referral trauma facility treating patients with polytrauma and high-energy injuries, may further limit generalizability. While statistical methods were used to reduce bias, controlling for confounding variables, such as comorbidities and patient-specific risk factors, remains challenging. Gender-specific differences observed in outcomes require further investigation into potential biological mechanisms. Future prospective, multi-center studies with standardized treatment protocols and longer follow-up periods are essential to validate these findings and enhance their clinical relevance.

### 2.7. Statistical Analysis

Descriptive statistics were utilized to summarize patient characteristics and outcomes. Chi-square tests (Pearson, NW, and Yates’ correction) assessed associations between categorical variables such as dislocation status, smoking status, fracture type, fixation type, and cause of reoperation, helping to reduce confounding effects. The Mann–Whitney U test compared continuous variables, such as observation time and age, between groups without assuming normal distribution. Receiver Operating Characteristic (ROC) curves were used to analyze statistically significant factors, evaluating their prognostic impact through the area under the curve (AUC) for robust analysis. To further control bias, stratified subgroup analysis was conducted to account for variables such as smoking, dislocation, and fixation type. Additionally, multivariable statistical adjustments were applied to enhance internal validity and mitigate confounding factors affecting reoperation rates and postoperative complications. Statistical significance was set at *p* < 0.05, and data analysis was performed using SPSS version 25 (IBM Corp., Armonk, NY, USA).

## 3. Results

### 3.1. Participants

The study included 246 patients, comprising 122 females and 124 males, ranging in age from 20 to 94 years, with a mean age of 51 years. Detailed patient characteristics are available in Table 1. During the study period, 42 patients required reoperation due to issues related to tibiofibular syndesmosis, with an average reoperation time of 30 weeks and a median of 23 weeks (ranging from 2 to 104 weeks). The average age of patients needing reoperation was 51 ± 16.8 years, with 56.67% non-smokers and 43.33% smokers. Among the 204 patients who did not require reoperation, 25.0% were smokers.

### 3.2. Dislocation and Reoperation

In the subgroup without dislocation, 16.28% required reoperation, while 83.72% did not. Among those with dislocation, 18.92% required reoperation, and 81.08% did not. A chi-square analysis revealed no statistically significant association between dislocation status and the necessity for reoperation (Pearson chi-square *p* = 0.61381).

### 3.3. Reoperation Causes and Combined Factors

Analyzing the interaction of factors such as smoking, dislocation, and fixation type revealed that smokers with dislocation had a higher rate of reoperation due to infection, while non-smokers without dislocation were primarily reoperated due to pain (persisting beyond six months postoperatively) (Table 2). One 59-year-old female non-smoker required reoperation after 8 months of observation due to nerve entrapment/inflammation. In her case, initial dislocation was absent, and the type B fracture was fixated with two screws. The detailed characteristics of patients who underwent reoperation for other reasons are provided in Table 3.

### 3.4. Observation Time and Reoperation Status

For the 42 patients who underwent reoperation, the mean observation time was 12.43 months (standard deviation (SD) = 15.60), with a median of 8.00 months. For those without reoperation, the mean observation time was 7.08 months (SD = 8.68), with a median of 5.00 months. The observation time for reoperated patients was significantly longer (*p* < 0.00001).

### 3.5. Time to Reoperation

The mean time to reoperation was 30.26 weeks (SD = 20.98), with a median of 23.00 weeks, indicating considerable variability and a wide range (2 to 104 weeks) in the onset of complications necessitating reoperation.

### 3.6. Age and Reoperation Status

The mean age of patients undergoing reoperation was 51.00 years (SD = 17.23), while for those not undergoing reoperation, it was 50.68 years (SD = 15.61), with no significant age difference between the two groups (*p* = 0.83).

### 3.7. Reoperation Causes

Kruskal–Wallis tests for observation time, time to reoperation, and age by reoperation cause did not yield significant results (*p* > 0.05), indicating no marked variation in these factors based on specific reoperation causes.

### 3.8. Type of Fracture and Postoperative Complications

Chi-square tests indicated no significant association between the fracture type and reoperation status (*p* > 0.05).

### 3.9. Fixation Type (Screw vs. Suturebutton) and Reoperation

No significant association was found between the type of fixation and reoperation status (*p* = 1.000).

### 3.10. Gender Differences in Outcomes

Reoperation rates between males and females were not statistically significant (*p* = 0.079), though there was a trend towards higher reoperation rates in males.

### 3.11. Impact of Smoking and Dislocation on Reoperation Rates

Patients with both smoking habits and dislocation had a significantly higher rate of reoperation compared to other groups. The chi-square test showed a Pearson chi-square value of 4.941 (*p* = 0.02622), indicating a statistically significant association between smoking, dislocation, and reoperation need. This effect was confirmed by additional chi-square statistics, including Yates’ correction and Fisher’s exact test, with *p*-values ranging from 0.04215 to 0.05502, reinforcing the significant impact of smoking and dislocation on reoperation risk.

### 3.12. Smoking as an Isolated Variable

When considered independently, smoking did not significantly impact reoperation rates. The Pearson chi-square test yielded a value of 0.233 (*p* = 0.62917), indicating no significant association. Other statistical tests corroborated this finding, with *p*-values consistently above 0.5.

### 3.13. Dislocation as an Isolated Variable

Dislocation alone also showed no significant correlation with reoperation rates (Pearson chi-square value of 0.254, *p* = 0.61381). Similar *p*-values from related tests supported this conclusion.

### 3.14. Combined Effect Analysis

The combined effect of smoking and dislocation revealed that patients with both factors had a significantly higher reoperation rate (35%) compared to those without these factors (15.49%). This suggests a synergistic effect, where the combination of smoking and dislocation exacerbates the risk of reoperation beyond the influence of each factor alone.

## 4. Discussion

We found that primary ankle fracture dislocation was not a negative prognostic factor for the surgical treatment of syndesmotic injuries, as dislocation alone did not show a significant correlation with reoperation rates.

In our study, all complications concerning the single four-cortex screw were caused by prolonged pain; no infectious complications or destabilizations were observed after this type of fixation. The single four-cortex screw might have provided adequate stabilization for the specific patient cohort, preventing mechanical complications such as screw loosening or fracture displacement. Despite these positive aspects, the prolonged pain experienced by patients suggests that, while the screw provided adequate stabilization and infection control, it may have caused local irritation, soft tissue impingement, or other biomechanical issues that led to persistent discomfort.

After suturebutton fixation, one complication was observed, i.e., the destabilization of the fixation, without pain symptoms. The fewest complications were observed in cases of isolated syndesmotic injury. The suture button system allows for some degree of natural movement, which can be beneficial for healing but might also lead to destabilization if the fixation is not secure enough. The absence of pain symptoms suggests that, while the fixation failed mechanically, it did not irritate surrounding tissues or cause significant inflammation. Isolated syndesmotic injuries typically involve less overall damage to the ankle joint and surrounding structures compared to more complex injuries. This means there is less overall stress and fewer forces acting on the fixation, leading to fewer complications.

In Weber C fractures, the destabilization of the fixation occurred most frequently. Most infectious complications occurred after fixation with a single three-cortex screw. Weber C fractures involve a higher location of the fibular fracture above the syndesmosis, often associated with more severe and unstable injuries. The increased mechanical forces and instability inherent in these fractures make successful fixation more challenging, leading to a higher likelihood of destabilization.

Statistically, men experienced the destabilization of the fixation and infectious complications more frequently, while women more commonly required surgery due to prolonged pain. Dislocations accounted for 42.8% of cases in the group with infectious complications; however, no significant statistical correlation was found. Smoking alone did not affect the frequency of reoperations, but smoking combined with dislocation significantly increased the frequency of reoperations. A total of 70% of patients who underwent reoperation due to infection had both dislocation and smoking habits, although this was not statistically significant. While smoking alone might not critically impair healing, its combination with the mechanical instability and tissue damage caused by dislocation exacerbates the healing process, leading to a higher need for reoperation. Smoking is known to impair wound healing due to its effects on blood circulation and oxygen delivery to tissues. When combined with the additional trauma and instability caused by dislocation, the compromised healing environment can lead to complications that necessitate reoperation. While the finding regarding the 70% infection rate was not statistically significant, it still indicates a clinically relevant trend. In clinical practice, understanding these trends can inform preventative measures and postoperative care strategies, even if statistical significance is not achieved in the study.

Overall, complications were predominantly influenced by the type of fixation, with men experiencing more frequent destabilization and infections, and smoking combined with dislocation significantly increasing reoperation rates.

We aimed to examine how initial dislocation affects complications after tibiofibular syndesmosis fixation and to explore how the fracture type and fixation method influence the frequency of complications and the need for reoperations. Ankle fracture dislocations are critical medical emergencies frequently addressed by foot–ankle surgeons, where delays in evaluating and realigning the ankle mortise can result in severe complications. Immediate reduction in ankle joint dislocations is essential as it helps to mitigate post-injury fatigue, prevent further damage to the articular cartilage, and lessen ankle pain [13]. Although several studies have investigated outcomes following syndesmotic injuries associated with unstable ankle fractures, the limited sample sizes and retrospective nature of these studies have restricted the understanding of the syndesmotic injury’s effect on patient functional outcomes [14]. Treating patients with ankle joint dislocations presents challenges due to potential ankle mortise deterioration and instability [15]. Some prior research has aimed to identify the prevalence of significant comorbidities post-surgery in patients with ankle dislocations and to analyze the relationship between trauma mechanisms, clinical status, and these comorbidities [16].

Birnie et al. investigated AITFL avulsion fractures in operatively treated ankle fractures. Conducted at a level-1 trauma center (2009–2017), this retrospective study included 252 patients (a mean age of 45 years, 52% male, and 58.3% right ankle fractures). Exclusion criteria were applied to filter out patients younger than 18, those without pre- and postoperative CT scans, and those with pilon fractures. AITFL avulsion fractures were present in 25.8% of patients. The distribution of Wagstaffe fracture types was 0% type 1, 43.1% type 2, 49.2% type 3, and 7.7% type 4. Additionally, 53.8% of the avulsed fragments were smaller than 5 mm, while 46.2% were 5 mm or larger. No significant differences in baseline characteristics were found between patients with and without AITFL avulsion fractures. Complications were more frequent in the non-fixated group, and three cases required revision surgery where a syndesmotic screw was initially used. The study questioned whether the direct fixation of the fragment would have prevented the need for revision surgery. Factors such as smoking, osteoporosis, and premature mobilization could have influenced the outcomes. The optimal treatment for AITFL avulsion fractures remains unclear due to the retrospective nature of the research [17].

Egol et al. evaluated the outcomes of operative treatment for unstable ankle fractures requiring syndesmotic stabilization (2000–2006). The study included 502 patients treated at a single institution by four fellowship-trained orthopedic traumatologists. Patients were followed at 3, 6, and 12 months postoperatively. Functional outcomes were measured using American Orthopaedic Foot & Ankle Society Scale (AOFAS) and Short Musculoskeletal Function Assessment (SMFA) tools, with clinical examinations and radiographic assessments to evaluate bony union, ankle mortise integrity, hardware condition, and the development of ankle arthrosis. Syndesmotic screws were not routinely removed unless necessary. The study found that adequate syndesmotic reduction was critical for better functional outcomes. Factors such as patient age, the number of screw cortices, medial malleolar fracture presence, and screw removal did not significantly impact results. The absence of radiographic malreductions supported the hypothesis that proper syndesmotic stabilization leads to better recovery, emphasizing the necessity of meticulous surgical techniques [14].

Yalın et al. assessed clinical and radiological outcomes in patients undergoing surgical treatment for ankle fracture dislocations, focusing on long-term ankle arthrosis. Thirty-eight patients were monitored for a minimum of 24 months postoperatively, with follow-up periods ranging from 24 to 48 months. Open fractures led to lower clinical and functional scores. Talar chondral lesions, often resulting from fracture dislocations, were significant precursors to arthrosis. The incidence of arthrosis was higher in older patients. Complications included superficial and deep tissue infections, with some requiring debridement or post-traumatic talectomy. Effective perioperative management was emphasized to mitigate complications and optimize outcomes. The study underscored the importance of recognizing risk factors such as patient age and talar chondral lesions for better prognostic assessments and patient counseling [15].

Andrés-Peiró et al., examined the predictors of postoperative complications following the fixation of low-energy ankle fractures in 663 patients. The study found a complication rate of 28.4%, with wound healing issues and infections predominant. Predictors included older age, longer surgery duration, and smoking. Each additional hour of surgery significantly increased complication risk, and smoking nearly tripled it. Tailored perioperative care for at-risk populations was highlighted to minimize complications [18].

Several studies identified negative prognostic factors in ankle fractures, including male gender, older age, Weber type C fractures, large tibiotalar dislocations in type B fractures, secondary osteosynthesis following temporary fixation, diabetes mellitus (DM), and a higher body mass index (BMI) [16,19,20]. Additional factors are high-energy trauma, open fractures, current smoking, alcoholism, and higher American Society of Anesthesiologists Physical Status Classification System (ASA) scores [16,21,22,23].

Marvan et al. studied distal tibiofibular synostosis development in surgically treated ankle fractures, including 824 patients with Weber type B and C fractures over nine years. Exclusions were made for associated fractures of the talus, calcaneus, and other foot bones. Synostosis occurred in 15.9% of patients, more commonly in men (21.1%) and older patients. Weber type C fractures had a higher incidence of synostosis (25.5%) than type B fractures (12.7%). Large tibiotalar dislocations (>10 mm) were significantly associated with synostosis in type B fractures, but not in type C fractures. Immediate definitive surgical treatment resulted in lower synostosis incidence (12.5%) compared to secondary osteosynthesis after temporary fixation (30.3%). The study concluded that male gender, older age, and certain fracture types are significant risk factors for synostosis, and immediate definitive surgical treatment may reduce its occurrence [19].

Lanzetti et al. examined wound complications in diabetic patients under 65 undergoing bimalleolar fracture surgery. Diabetics had higher complication rates (38.9%) compared to non-diabetics. Wound complications included infections, delayed healing, deep vein thrombosis, and pulmonary embolism. DM increased the likelihood of wound complications by 4.33 times. A higher BMI also significantly increased the risk of postoperative complications. Despite complications, all fractures healed within five months. The study emphasized meticulous perioperative glycemic control in diabetic patients to mitigate complications. Prolonged hyperglycemia impairs wound healing through vasculopathy and neuropathy, increasing susceptibility to infections and delayed healing [20].

Testa et al. evaluated trimalleolar fracture treatment outcomes, noting better recovery in younger patients with normal weight and lower ASA classification. Poor outcomes were associated with advanced age, high BMI, and severe fracture types. Functional outcomes were assessed using the Olerud–Molander scale (OMAS) score and the Visual Analog Scale (VAS) for pain. Type C fractures or fracture dislocations reported lower OMAS scores and higher VAS scores, indicating worse functional outcomes and greater residual pain. Surgical treatment generally led to favorable outcomes, with 17% reporting excellent, 62% good, and 23% poor outcomes [21].

Xiao et al. identified risk factors for postoperative wound complications in ankle fracture surgery and analyzed clinical data from 200 patients treated at Beijing TongRen Hospital (2021–2023). Postoperative wound complications occurred in 19% of patients. Significant risk factors included age, BMI, smoking, alcoholism, DM, injury mechanism, open fractures, wound classification, and higher ASA scores. Multivariate logistic regression identified nine independent risk factors, with age over 60 years (OR = 3.671), higher BMI (OR = 1.198), current smoking (OR = 2.727), alcoholism (OR = 1.143), DM (OR = 2.763), high-energy injury mechanism (OR = 2.437), open fractures (OR = 1.943), wound classification II versus I (OR = 4.423), and ASA score III–IV versus I–II (OR = 1.307). ROC curves showed high accuracy and validity in predicting postoperative wound complications, emphasizing the need for tailored perioperative care [16].

Jerjes et al. investigated the impact of chronic heavy tobacco smoking on healing outcomes of ankle fractures. This comparative analysis included 220 chronic heavy smokers and an equal number of non-smokers. Smokers experienced significant delays in fracture union and higher postoperative complications, such as prolonged pain, swelling, and infections. Superficial and deep wound infections were more common in smokers. The study highlighted the detrimental effects of smoking on bone and soft tissue healing following surgical interventions [22].

Smoking-induced oxidative stress disrupts bone turnover by increasing reactive oxygen species (ROS), which enhance osteoclast activity, inhibit osteoblast function, and accelerate osteocyte apoptosis. This imbalance leads to reduced bone mineral density, osteoporosis, delayed fracture healing, and increased fracture risk. Additionally, smoking disrupts collagen synthesis, further impairing bone structure [24]. Oxidative stress favors bone resorption, but antioxidants can counteract these effects by promoting osteoblast differentiation, enhancing mineralization, and reducing osteoclast activity. Clinical evidence links low antioxidant levels to osteoporosis, suggesting that antioxidant-based therapies, such as polyphenols and vitamins, may help mitigate bone loss [25].

Lu et al. demonstrated that cigarette smoke exposure disrupts bone remodeling by increasing osteoclast activity and inhibiting osteoblast differentiation. Smoking-induced oxidative stress, mediated by NFκB activation, enhances bone resorption and reduces bone formation, leading to decreased bone mineral density and increased fracture risk. Chronic exposure further amplifies these effects, contributing to osteoporosis and delayed fracture healing. The findings highlight the critical role of inflammation in smoking-related bone loss and suggest that targeting NFκB signaling could mitigate these adverse effects [26].

Dabert et al. investigated bone consolidation outcomes under septic conditions while retaining internal fixation hardware. The study was conducted at a single center (2009–2019), and it included 69 patients with bone and joint infections diagnosed based on Musculoskeletal Infection Society (MSIS) criteria. Bone healing occurred in 73.5% of patients, with a mean consolidation time of 24 weeks. Timing of surgical intervention did not significantly affect the consolidation rate. Key risk factors for non-consolidation included smoking, a longer initial surgery time, and severe fractures (Gustilo type IIIb or IIIc). The study supported that bone consolidation can be achieved under septic conditions with appropriate management, including thorough debridement and irrigation. Smoking was a significant risk factor, corroborating previous findings [23].

Marais et al. emphasized the importance of early surgical intervention in fracture-related infections to prevent persistent infections, ensure proper fracture healing, and reduce complications. Surgical strategies include implant retention, revision, or removal, with the decision based on the bone condition, soft tissues, host factors, and the causative microorganism. Urgent surgery is recommended for progressive infections, while elective procedures allow for patient optimization. Effective management requires a multidisciplinary approach, combining surgical debridement, fracture stabilization, and antibiotic therapy. Standardized treatment protocols and further research are needed to refine management strategies and improve patient outcomes [27].

Goswami et al. explored the role of biofilms in chronic wound healing, emphasizing their resistance to antibiotics and contribution to prolonged inflammation. Biofilms protect bacteria from immune responses, leading to persistent infections and delayed wound closure. Current treatment strategies include mechanical debridement, antimicrobial agents, and novel biofilm-targeting therapies. However, standardized clinical protocols are lacking, and laboratory findings often fail to translate into effective bedside treatments. The study highlights the need for further research to develop clinically viable anti-biofilm strategies, improve wound care, and enhance patient outcomes by integrating biofilm disruption techniques into routine clinical practice [28].

Domzalski et al. examined the impact of smoking on knee function and recovery following meniscus repair using the outside-in technique. This retrospective cohort study reviewed 99 patients, with 92 available for follow-up, categorized into smokers and non-smokers. Smokers had significantly worse knee function (average Knee Injury and Osteoarthritis Outcome Score (KOOS) score 67.4 vs. 80.2) and longer recovery times for daily activities (5.4 months vs. 4.2 months) and sports (9.4 months vs. 7.6 months). Statistical analysis confirmed smoking was significantly associated with delayed recovery and reduced knee function post-surgery. The study controlled for variables like age and BMI, attributing the detrimental effects to smoking. Results underscored the negative influence of smoking on tissue healing, involving factors like reduced perfusion, impaired cell function, and diminished collagen synthesis [29].

## 5. Conclusions

The study identifies several factors influencing the prognosis of surgical treatment for syndesmotic injuries, emphasizing the complexity of managing these injuries. Immediate reduction in dislocated ankles is crucial, with external fixation reserved for cases with severe soft tissue damage. While initial dislocation alone was not significantly associated with reoperation rates, the combination of smoking and dislocation notably increased the risk of complications requiring reoperation. The type of fixation method and patient-specific factors such as age, gender, and smoking status played critical roles in determining outcomes. Particularly, men and smokers with dislocated fractures experienced higher rates of reoperation due to complications like infection and destabilization. These findings highlight the need for meticulous surgical techniques, personalized treatment plans considering patient demographics and comorbidities, and rigorous postoperative monitoring to optimize outcomes. Future prospective studies with larger sample sizes and standardized protocols are needed to validate these findings and further refine treatment strategies for syndesmotic injuries.

## Figures and Tables

**Table 1:** Patient characteristics.

Factor		Study Group [*n* = 246]		No Reoperation [*n* = 204]		Reoperation [*n* = 42]		*p*
		N	%	N	%	N	%	
Fracture type	Syndesmosis	15	6.10%	14	6.86%	1	2.38%	0.514
	Weber B	170	69.11%	139	68.14%	31	73.81%	
	Weber C	61	24.80%	51	25.00%	10	23.81%	
	Synostosis	15	6.10%	14	6.86%	1	2.38%	0.27
	Weber B + C	231	93.90%	190	93.14%	41	97.62%	
Synthesis type	Endobutton	19	7.72%	18	8.82%	1	2.38%	0.436
	2 screws	52	21.14%	41	20.10%	11	26.19%	
	1 three-cortical screw	134	54.47%	110	53.92%	24	57.14%	
	1 four-cortical screw	41	16.67%	35	17.16%	6	14.29%	
Reoperation * cause	Pain	22	8.94%	-	-	22	52.38%	-
	Destabilization	5	2.03%	-	-	5	11.90%	
	Infection	14	5.69%	-	-	14	33.33%	
	Nerve entrapment/inflammation	1	0.41%	-	-	1	2.38%	
Reoperation * type	Screw removal	14	5.69%	-	-	14	33.33%	-
	Other ^#^	28	11.38%	-	-	28	66.67%	
Dislocation	No	172	69.92%	144	70.59%	28	66.67%	0.613
	Yes	74	30.08%	60	29.41%	14	33.33%	
Gender	Male	124	50.41%	101	49.51%	23	54.76%	0.535
	Female	122	49.59%	103	50.49%	19	45.24%	
Age (years)	20–40	66	26.83%	57	27.94%	9	21.43%	0.432
	41–60	107	43.50%	85	41.67%	22	52.38%	
	>60	73	29.67%	62	30.39%	11	26.19%	

* A surgical procedure to address complications related to syndesmotic fixation, including the removal of the fixation for pain or infection or replacement in cases of destabilization. ^#^ Various additional surgical interventions, such as fixation replacement, debridement, peroneal nerve release, or the complete removal of the fixation system.

**Table 2:** Reoperation causes by smoking status (*n* = 42).

Reoperation Causes by Smoking Status		
Cause of Reoperation	Non-Smokers (%)	Smokers (%)
Pain	56.67	41.67
Destabilization	16.67	0.00
Infection	23.33	58.33
Nerve entrapment/inflammation	3.33	0.00

**Table 3:** Characteristics of patients who underwent reoperation due to pain, destabilization, or infection (*n* = 41).

Factor		Pain (%)	Destabilization (%)	Infection (%)
**Gender**	Male	34.78	20.00	78.57
	Female	65.22	80.00	21.43
Observation time (months)	<24	20.00	20.00	14.29
	24–48	30.00	30.00	35.71
	>48	50.00	50.00	50.00
Age group (years)	20–40	20.00	60.00	14.29
	41–60	50.00	20.00	50.00
	61+	30.00	20.00	35.71
Smoking and dislocation	Smokers with dislocation	57.14	0.00	42.86
	Non-smokers with dislocation	44.44	22.22	33.33
	Smokers without dislocation	25.00	0.00	75.00
	Non-smokers without dislocation	66.67	16.67	16.67
Type of fracture	Weber B	78.26	20.00	85.71
	Weber C	21.74	60.00	14.29
	Synostosis	0.00	20.00	0.00
Synthesis type	Endobutton	0.00	20.00	0.00
	2 screws	26.09	40.00	21.43
	1 screw three-cortical	47.83	40.00	78.57
	1 screw four-cortical	26.09	0.00	0.00

## Data Availability

The data presented in this study are available on request from the corresponding author (BGW).

## Abbreviations

The following abbreviations are used in this manuscript:

AITFL	anterior inferior tibiofibular ligament
PITFL	posterior inferior tibiofibular ligament
ORIF	open reduction and internal fixation
CAM	Controlled Ankle Motion
CT	Computed Tomography
AUC	area under the curve
ROC	Receiver Operating Characteristic
SD	standard deviation
AOFAS	American Orthopaedic Foot & Ankle Society Scale
SMFA	Short Musculoskeletal Function Assessment
DM	diabetes mellitus
BMI	Body Mass Index
ASA	American Society of Anesthesiologists Physical Status Classification System
O&M	Olerud–Molander score
VAS	Visual Analog Scale
ROS	Reactive oxygen species
MSIS	Musculoskeletal Infection Society
KOOS	Knee Injury and Osteoarthritis Outcome Score

## References

[1] Herzog M.M., Kerr Z.Y., Marshall S.W., Wikstrom E.A. (2019). Epidemiology of ankle sprains and chronic ankle instability. J. Athl. Train..

[2] Doherty C., Delahunt E., Caulfield B., Hertel J., Ryan J., Bleakley C. (2014). The incidence and prevalence of ankle sprain injury: A systematic review and meta-analysis of prospective epidemiological studies. Sports Med..

[3] Bejarano-Pineda L., Guss D., Waryasz G., DiGiovanni C.W., Kwon J.Y. (2021). The syndesmosis, Part I: Anatomy, injury mechanism, classification, and diagnosis. Orthop. Clin. N. Am..

[4] Liu J., Valentine D., Ebraheim N.A. (2022). Management of syndesmosis injury: A narrative review. Orthop. Res. Rev..

[5] Sanders F.R.K., Birnie M.F.N., Dingemans S.A., van den Bekerom M.P.J., Parkkinen M., van Veen R.N., Goslings J.C., Schepers T., RODEO Collaborator Group (2021). Functional outcome of routine versus on-demand removal of the syndesmotic screw: A multicentre randomized controlled trial. Bone Jt. J..

[6] Lee J.S., Curnutte B., Pan K., Liu J., Ebraheim N.A. (2021). Biomechanical comparison of suture-button, bioabsorbable screw, and metal screw for ankle syndesmotic repair: A meta-analysis. Foot Ankle Surg..

[7] Liu J., Pathak G., Joshi M., Andrews K., Lee J. (2021). A meta-analysis comparing the outcomes of syndesmotic injury treated with metal screw, dynamic fixation, and bioabsorbable screw. J. Orthop..

[8] Ostrum R.F., Avery M.C. (2016). Open reduction internal fixation of a bimalleolar ankle fracture with syndesmotic injury. J. Orthop. Trauma.

[9] Glen L.Z.Q., Wong J.Y.S., Tay W.X., Li T.P., Phua S.K.A., Manohara R., Chee Y.H. (2023). Weber ankle fracture classification system yields greatest interobserver and intraobserver reliability over AO/OTA and Lauge-Hansen classification systems under time constraints in an Asian population. J. Foot Ankle Surg..

[10] Stolycia M.L., Lunn D.E., Stanier W., Walker J., Wilkins R.A. (2024). Biomechanical effectiveness of controlled ankle motion boots: A systematic review and narrative synthesis. J. Foot Ankle Res..

[11] Davey M.S., Davey M.G., Hurley E.T., Kearns S.R. (2022). Tourniquet use during open reduction and internal fixation of ankle fractures—A systematic review and meta-analysis. J. Foot Ankle Surg..

[12] Tantigate D., Ho G., Kirschenbaum J., Bäcker H., Asherman B., Freibott C., Greisberg J.K., Vosseller J.T. (2019). Timing of open reduction and internal fixation of ankle fractures. Foot Ankle Spec..

[13] Schepers T., De Vries M.R., Van Lieshout E.M., Van der Elst M. (2013). The timing of ankle fracture surgery and the effect on infectious complications: A case series and systematic review of the literature. Int. Orthop..

[14] Egol K.A., Pahk B., Walsh M., Tejwani N.C., Davidovitch R.I., Koval K.J. (2010). Outcome after unstable ankle fracture: Effect of syndesmotic stabilization. J. Orthop. Trauma.

[15] Yalın M., Aslantaş F.Ç., Duramaz A., Bilgili M.G., Baca E., Koluman A. (2020). The common comorbidities leading to poor clinical outcomes after the surgical treatment of ankle fracture-dislocations. Ulus Travma Acil Cerrahi Derg..

[16] Xiao B., Lu M., Chen X., Qiu D., He Y., Li X. (2024). Study on the risk factors of postoperative wound complications in patients with ankle fracture. Int. Wound J..

[17] Birnie M.F.N., van Schilt K.L.J., Sanders F.R.K., Kloen P., Schepers T. (2019). Anterior inferior tibiofibular ligament avulsion fractures in operatively treated ankle fractures: A retrospective analysis. Arch. Orthop. Trauma Surg..

[18] Andrés-Peiró J.V., Pujol O., Altayó-Carulla M., Castellanos-Alonso S., Reverté-Vinaixa M.M., Teixidor-Serra J., Tomàs-Hernández J., Selga-Marsà J., García-Sánchez Y., Molero-GarcIa V. (2024). Predictors of first-year postoperative complications after fixation of low-energy ankle fractures: A single-center, retrospective cohort study of 663 consecutive fractures. Rev. Esp. Cir. Ortop. Traumatol..

[19] Marvan J., Ježek J., Vránová J., Marvan D., Čížek F., Džupa V. (2022). Risk factors for the development of distal tibiofibular synostosis after unstable ankle fractures. Acta Chir. Orthop. Traumatol. Cech..

[20] Lanzetti R.M., Lupariello D., Venditto T., Guzzini M., Ponzo A., De Carli A., Ferretti A. (2018). The role of diabetes mellitus and BMI in the surgical treatment of ankle fractures. Diabetes Metab. Res. Rev..

[21] Testa G., Ganci M., Amico M., Papotto G., Giardina S.M.C., Sessa G., Pavone V. (2019). Negative prognostic factors in surgical treatment for trimalleolar fractures. Eur. J. Orthop. Surg. Traumatol..

[22] Jerjes W., Ramsay D., Stevenson H., Yousif A. (2024). Effect of chronic heavy tobacco smoking on ankle fracture healing. Foot Ankle Surg..

[23] Dabert A., Runtz A., Leclerc G., Sergent P., Loisel F., Pluvy I., Fradin T., Garbuio P. (2024). Bone consolidation under septic condition and hardware retention: About 69 patients. Orthop. Traumatol. Surg. Res..

[24] Kohler J.B., Junqueira J.J.M., Silva T.C.M.D., Filho M.A.G.P., Tibério I.D.F.L.C., Lopes F.D.T.Q.S., Barbosa A.P. (2021). Smoking-induced oxidative stress in bone: The effects on bone turnover. J. Orthop. Orthop. Surg..

[25] Domazetovic V., Marcucci G., Iantomasi T., Brandi M.L., Vincenzini M.T. (2017). Oxidative stress in bone remodeling: Role of antioxidants. Clin. Cases Miner. Bone Metab..

[26] Lu Y., Di Y.P., Chang M., Huang X., Chen Q., Hong N., Kahkonen B.A., Di M.E., Yu C., Keller E.T. (2021). Cigarette smoke-associated inflammation impairs bone remodeling through NFκB activation. J. Transl. Med..

[27] Marais L.C., Zalavras C.G., Moriarty F.T., Kühl R., Metsemakers W.J., Morgenstern M. (2023). The surgical management of fracture-related infection. Surgical strategy selection and the need for early surgical intervention. J. Orthop..

[28] Goswami A.G., Basu S., Banerjee T., Shukla V.K. (2023). Biofilm and wound healing: From bench to bedside. Eur. J. Med. Res..

[29] Domzalski M., Muszynski K., Mostowy M., Wojtowicz J., Garlinska A. (2021). Smoking is associated with prolonged time of the return to daily and sport activities and decreased knee function after meniscus repair with outside-in technique: Retrospective cohort study. J. Orthop. Surg..

